# Current Approaches to Low Vision (Re)Habilitation

**DOI:** 10.4274/tjo.galenos.2018.53325

**Published:** 2019-06-27

**Authors:** Deniz Altınbay, Şefay Aysun İdil

**Affiliations:** 1 Private Niv Eye Center, Ophthalmology Clinic, Adana, Turkey; 2 Ankara University, Artificial Vision and Low Vision Rehabilitation, Master Student with Thesis in Vision, Ankara, Turkey; 3 Ankara University Faculty of Medicine, Department of Ophthalmology, Ankara, Turkey; 4 Center of Vision Research and Low Vision Rehabilitation, Ankara, Turkey

**Keywords:** Low vision, low vision (re)habilitation, Current approaches, LVA

## Abstract

With increased life expectancy at birth and especially the rising incidence of age-related macular degeneration, low vision (re)habilitation is becoming more important today. Important factors to consider when presenting rehabilitation and treatment options to patients presenting to low vision centers include the diagnosis of the underlying disease, the patient’s age, their existing visual functions (especially distance and near visual acuity), whether visual loss is central or peripheral, whether their disease is progressive or not, the patient’s education level, and their expectations from us. Low vision patients must be guided to the right centers at the appropriate age, with appropriate indications, and with realistic expectations, and the rehabilitation process must be carried out as a multidisciplinary collaboration.

## Introduction

Visual impairment in low vision (re)habilitation may be central or peripheral vision loss or reduced vision due to media opacity. Among these groups, the most common diagnosis in patients presenting to low vision clinics is age-related macular degeneration (AMD), which causes central vision loss.^1,2,3,4,5,6,7^

The type of rehabilitation required by the low vision patient varies depending on their visual acuity, age, sociocultural status, and especially their diagnosis. The approach to a patient who has central scotoma due to AMD is quite different from the approach to a patient who has tunnel vision due to retinitis pigmentosa. Some cases can involve the coexistence of both central and peripheral vision loss, as in the patient with concurrent diabetic maculopathy and diabetic retinopathy who underwent argon laser treatment to the peripheral retina.

The aim of low vision rehabilitation is for patients to use their residual vision as effectively and efficiently as possible to enable them to live as self-sufficient, independent, and productive individuals, to make their lives easier, and enhance their quality of life. Low vision rehabilitation is not limited to simply recommending aids such as telescopic glasses or magnifying glasses. More important are training in the use these devices and the rehabilitation process. Rehabilitation is a collaborative effort involving many professional groups, such as vocational therapists, psychologists, and social workers, led by an ophthalmologist.

The Vision Research and Low Vision Rehabilitation Center of the Department of Ophthalmology of Ankara University Faculty of Medicine is the first vision rehabilitation center in Turkey to be established within the body of a university, and has facilitated the rehabilitation of 5500 individuals with low vision to date. The center also runs a thesis master’s program on the subject for ophthalmologists.

### What are the Current (Re)Habilitation/Treatment Methods for Low Vision?

- Field expansion prisms for peripheral visual field loss,

- Microperimetry,

- Telescopic intraocular lenses,

- Telescopic contact lenses,

- Argus II epiretinal prosthesis (bionic eye),

- BrainPort,

- Stem cell therapy,

- Platelet-rich plasma (PRP) and electrical stimulation,

- Gene therapy.

### Prisms for Field Expansion in Patients with Peripheral Vision Loss

Magnification is the main objective in the aid and rehabilitation of low vision patients. An object is enlarged and/or zoomed into. This method provides satisfactory results in the rehabilitation of patients with central visual field loss, especially for reading. However, in patients with peripheral vision loss (PVL), as in retinitis pigmentosa and glaucoma, magnification may further reduce existing vision instead of being helpful if the patient’s visual field has become too narrow. In this case, telescopes that expand the visual field (reverse telescopes) can be used. However, this will decrease the patient’s visual acuity. A 0.5X telescope increases a patient’s visual field by 2 fold, but also decreases their visual acuity by half, and this method is therefore not highly preferred by patients.

**Figure 1:**
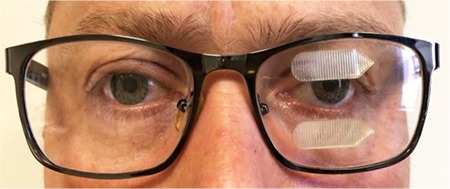
The ML Peli Prism/Multilens field expansion Peripheral Fresnel prism (from the archive of Prof. Şefay Aysun İdil, MD)

The use of field expansion prisms is more appropriate than telescopes in patients with PVL. Peli’s field expansion prisms can be used in patients who have homonymous hemianopsia due to neurological causes. In such cases, prisms are placed on the affected side with the base toward the side of the field defect (e.g., on the left eye with the base facing outward for left-sided homonymous hemianopsia). The prisms are monocular and are placed on the posterior surface of the spectacle lens in the upper and lower quadrant with a central opening between them, bases facing the defect. The central opening is 12 mm. There are horizontal and oblique varieties (Figure 1, oblique peli prism). These high-diopter (D) prisms expand the patient’s visual field in the direction of the field defect. After the initial application of Fresnel prisms, the patient is given training exercises. If the patient is comfortable and adapted to the visual field expansion, the prisms are permanently attached to the lens.^8^ These prisms are used at our center.

In a patient with left-sided hemianopsia, a 40Δ D horizontal prism placed base-out over the left eye provides a field expansion of 20 degrees, while a 40Δ D oblique prism with upper segment base out and down and lower segment base out and up provides a field expansion of 30 degrees.

Patients with tunnel vision are also a challenging group in low vision rehabilitation. Especially in diseases like retinitis pigmentosa and choroideremia, patients can have PVL in all quadrants. In such cases, patients may be recommended a Trifield prism. Trifield prisms are monocular and placed base-out in the temporal quadrant and base-in in the nasal quadrant of the spectacle lens, and the other eye provides central vision. Three fields are available to the patient and field expansion is provided in all directions of view. Training is very important. The prisms are colored to reduce double vision and confusion.^9^

These field expansion prisms provide awareness of the absent field, but cannot treat visual field losses.^10^

### Microperimetry

Since traditional visual field tests are based on the premise that the patient has central and stable fixation during the test, their reliability is reduced for patients with macular disease who have extrafoveal and/or unstable fixation and whose central vision is primarily affected. Standard visual field testing is also unable to detect small scotomas or provide reliable results in patients with very low vision. Therefore, traditional visual field tests remain inadequate for patients with macular disease. Obtaining reliable test results from macular sensitivity measurements is difficult in patients with advanced macular disease due to unstable fixation.^11,12^ Microperimetric examination has been shown to enable assessment of retinal sensitivity as well as fixation characteristics, even in patients with severe visual impairment.^13^

Microperimetry is as valuable as standard visual field testing for demonstrating retinal sensitivity, and superior to standard visual field tests for demonstrating the early stages of vision loss.^14,15^

By superimposing visual field test results on fundus images, the microperimetry device allows morphological and functional examination to be performed together. It can also determine scotoma location and the location and stability of fixation in patients with macular disease. It can show retinal sensitivity in the target retinal area in decibels (dB) numerically, schematically, or on a color scale. A reference point is marked on an infrared image taken at the start of acquisition, and visual field results are superimposed on a color fundus image taken after the procedure to demonstrate the relationship between the scotoma and macular pathology. With the eye tracking system of the microperimetry device, even if the patient’s fixation characteristics change over the course of follow-up, measurements in later scans can be made from the reference points marked in the initial reading, thus ensuring reliability of the results.

AMD is the leading cause of severe visual impairment and legal blindness in developed countries, especially in those aged 65 years and older. Central scotomas in the advanced stage cause central vision loss and limit capacity to perform daily activities, decreasing patients’ quality of life. Impairment of visual function in AMD has been demonstrated in microperimetry as reduction in fixation stability, loss of central fixation, and loss of retinal sensitivity.^16^ In these patients, the nonfunctional fovea is replaced by eccentric locations in healthier retinal regions, called the preferred retinal locus (PRL). Fixation characteristics and the PRL are of great importance in patients with central scotomas in terms of ability to perform activities of daily living. This area can be detected by microperimetry. Determining scotoma size and location and knowing the location and stability of fixation are essential for low vision rehabilitation.

In some patients, the PRL is not in an appropriate place, and must be moved to a location that is more suitable for the patient and has higher retinal sensitivity. Using the biofeedback feature of the microperimetry device, this area can be relocated to healthier retinal regions with PRL shifting exercises (trained retinal locus, TRL).^17^

Approximately 60% of patients referred to low vision centers present due to difficulty reading. Fixation stability and location are among the factors that most affect a patient’s vision quality and reading performance in particular. A study by Giacomelli et al.^18^ including diabetic retinopathy and AMD patients with mild to moderate low vision (0.3-1.0 LogMAR) showed that fixation instability and loss of contrast sensitivity were the factors that most affected reading performance. In another study, a strong correlation was detected between fixation stability and reading speed.^19^

In this patient group, monitoring and rehabilitation carried out with the microperimetry device will improve reading performance and may thereby improve the patients’ quality of life.

Microperimetry is used not only in patients with low vision due to AMD, but also for the rehabilitation of patients with low vision due to causes such as retinitis pigmentosa, Stargardt disease, diabetic retinopathy, and glaucoma. Microperimetry has also been reported to provide valuable information on macular function in cases of ABCA4-associated retinal degenerative diseases (Stargardt disease and cone-rod dystrophy) and night blindness.^20^

### Parameters Evaluated by Microperimetry

**PRL-high:** The center of the points obtained while focusing on the fixation point in the first 10 seconds, before stimulus presentation.

**PRL-low:** The center of all fixation points calculated at the end of the testing period.

P1 and P2 are the proportions of fixation points within 1° and 2° areas, respectively.

**Fixation stability:** P1>75% indicates stable fixation, P1<75% and P2>75% indicate relatively stable fixation, and P2<75% indicates unstable fixation.

**Fixation location:** More than 50% of fixation points falling within the central standard fixation area is classified as predominantly central fixation, 25-50% within the central standard fixation area as weak central fixation, and less than 25% being within the central standard fixation area as predominantly eccentric fixation.

**Macular integrity index (MII):** Provides age-matched average data. Loss is considered normal if less than 40%, suspicious if 40-60%, and abnormal if above 60%.

Average retinal sensitivity: Results range from 0 dB to 36 dB. Values of 0-23 dB are considered normal, 23-25 dB suspicious, and 25-36 dB abnormal.

**BCEA (bivariate contour ellipse area):** Indicates the elliptical area of major and minor axes covered by fixational eye movements.

**Figure 2:**
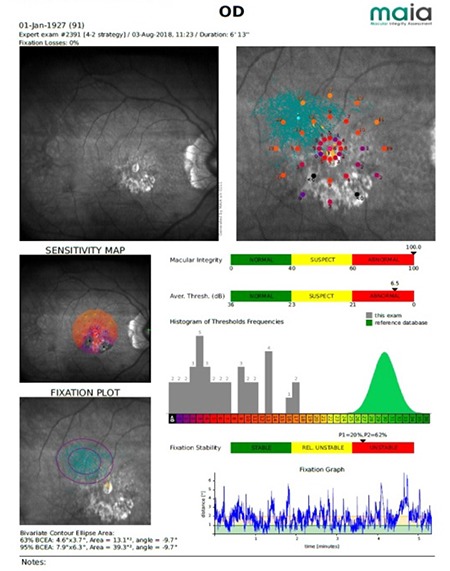
Sample microperimetry output (from the archive of Prof. Şefay Aysun İdil, MD) OD: Right eye

These parameters are shown in the device’s output (Figure 2).

### Interpretation of Microperimetry Results (Figure 2)

- Right eye, 91-year-old atrophic AMD patient,

- PRL is located in the superotemporal aspect of the atrophic site and retinal sensitivity is 11–17 dB in this region,

- Mode: Expert Test, Strategy: 4-2,

- Thirty-seven points, central 10°,

- Average sensitivity: 6.5 dB,

- MII: 100,

- Fixation Stability: Unstable (P1=20%, P2=62%),

- BCEA: 63% = 4.6°x3.7°, 13.1°^2^ BCEA: 95% = 7.9°x6.3°, 39.3°^2^,

- Fixation location (PRL): Superotemporal,

 - Test duration: 6’13”,

- Central scotoma, fixation is unstable and extrafoveal.

In macular diseases, microperimetry reveals reduced fixation stability, loss of central fixation, and loss of retinal sensitivity. In this example from a patient with AMD, it can be seen that there is a decrease in fixation stability (P1=20%, P2=62%), loss of central fixation (superotemporal fixation), and severe loss of retinal sensitivity (average 6.5 dB).

**Figure 3:**
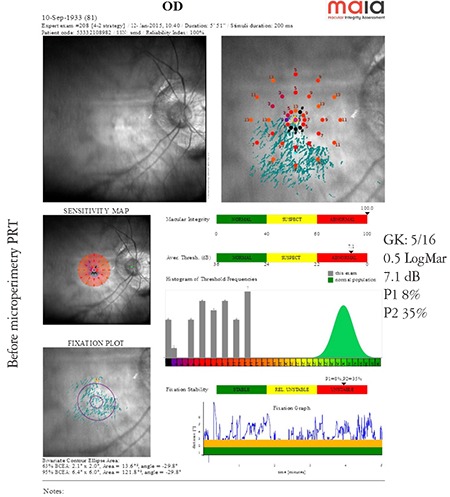
Microperimetry values of a patient with macular disease with unstable fixation before preferred retinal locus training (from the archive of Prof. Şefay Aysun İdil, MD) OD: Right eye

**Figure 4:**
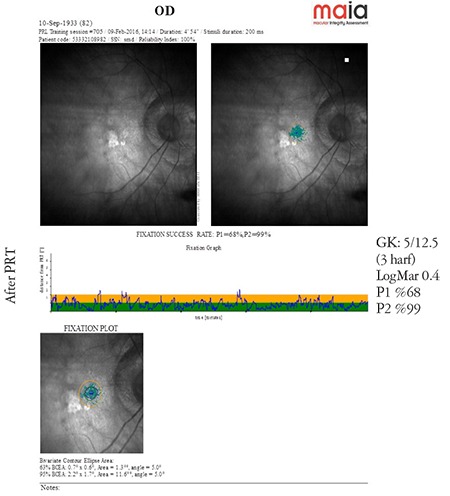
Microperimetry shows increased stability in the same patient after preferred retinal locus training (from the archive of Prof. Şefay Aysun İdil, MD) OD: Right eye

**Microperimetry TRL (trained retinal locus) mode:** The microperimetry TRL mode improves the stability of the PRL formed by the patient if its location is favorable. The microperimetry readings of a macular disease patient with an unstable PRL (P1 8%, P2 35%) obtained before and after PRL training are shown in Figures 3 and 4. Comparison of the microperimetry readings demonstrate a remarkable increase in the stability of the patient’s PRL (P1: 68%, P2: 99%, relatively stable PRL) (Figures 3 and 4).

If the location of the patient’s PRL is unfavorable, it is shifted to an area more appropriate for the patient. The PRL Training mode helps patients with low vision, especially those with a central scotoma and unstable fixation, to better utilize their residual vision with auditory and visual biofeedback signals and eccentric viewing therapy. When choosing a new PRL, the area closest to the fovea and the patient’s existing PRL and with the highest retinal sensitivity should be selected.

The purpose of using microperimetry in low vision rehabilitation is to help the low vision patient use their residual vision as efficiently as possible. In rehabilitation, the aim is to use the microperimetry device to enhance fixation stability if the patient’s PRL is in a suitable location but is not stable enough or if the PRL is not in a suitable location, to identify and relocate the PRL to a locus with higher retinal sensitivity through TRL training sessions.

### Telescopic Intraocular Lenses

With recent advances in technology and subsequently in intraocular lenses, attempts have been made to provide magnification in low vision patients with AMD via surgical methods.

To date, seven types of intraocular lenses have been used in patients with AMD. None of the current telescopic lenses are ideal, and only short-term results have been published. These include the implantable miniature telescope (IMT), IOL-VIP System, Lipshitz macular implant (LMI), sulcus-implanted Lipshitz macular implant (LMI-SI), Fresnel prism intraocular lens, iolAMD, and Scharioth Macula Lens. The magnification power of the lenses are as follows: 1.2X with the iolAMD lens, 2.5X with the IMT, 1.3X with the IOL-VIP system, 2.5X with the LMI, and 1X in the Fresnel prism intraocular lens.

The IMT is larger than the other implantable telescopic lenses and requires a large incision. There may be some difficulties in fundus imaging after implantation.^21^

The LMI and LMI-SI utilize lenses with two miniature mirrors in a Cassegrain telescope configuration and magnify the image reflected on the retina 2.5 times.^22^ There may be difficulties in fundus imaging due to glare. While the LMI is implanted in the capsular bag, the LMI-SI can be implanted in the sulcus in pseudophakic patients.

The aim of Fresnel prism intraocular lenses is not magnification, but rather to shift the position of the scotoma. A Fresnel prism is present on the rear surface of the optical part of the lens.^23^

The iolAMD is acrylic and aims to create a Galilean telescopic effect using -49 D and +63 D lenses. The disadvantage of this lens is that its power cannot be adjusted according to the axial length of the eye.^24^

**IOL-VIP system telescopic intraocular lenses:** The IOL-VIP system uses -66 D biconcave and +55 D biconvex lenses and provides 1.3X magnification. Simulation should be performed prior to surgery. With the IOL-VIP Revolution, two lenses are placed in the capsule with a tension ring to create a telescopic effect. At the same time, the intention is to shift the image from the diseased retina to the healthier retinal area via prismatic effect (about 10 prism D). The visual rehabilitation process is complex.^25^

### Indications

- Atrophic AMD,

- Visual acuity lower than 0.3,

- Visual acuity is enhanced by a simulator,

- Patient willingness,

- After completion of a rehabilitation program (6 weeks).

### Contraindications

- Exudative AMD,

- Progressive visual field loss, as in glaucoma, retinitis pigmentosa, and diabetic retinopathy,

- Presence of corneal guttata, endothelial cell count less than 1600,

- Microphthalmia,

- Vision is not enhanced by an external simulator,

- Young patients (power of accommodation is lost postoperatively).

**Figure 5:**
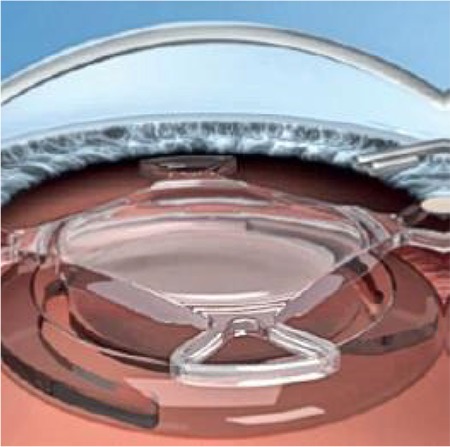
Scharioth macula lens (from the KMDT [Kesin Distribution and Foreign Trade Co. Ltd.] and Medicontur Turkey representative brochure)

**Scharioth macula lens (SML) telescopic intraocular implant: **These are used in pseudophakic patients. They are acrylic, and feature a +10.00 addition in the center of the lens (Figure 5).^26^The goal is to facilitate near reading. The SML enables near distance reading without distorting distance vision. The patient should be informed before the operation that they will have a short reading distance (10-15 cm) postoperatively. In a study presenting the 6-month results of 8 patients who received SML implants, it was reported that patients had difficulties with reading speed and reading distance that improved with reading exercises, and atrophic AMD progressed to wet AMD in 1 of the 8 patients at postoperative 3 months.^27^

### Indications

- Pseudophakic patients over 55 years of age,

- Visual acuity ≤0.32,

- Visual acuity increases >3 rows when reading from a distance of 15 cm with a +6.00 addition preoperatively,

- Atrophic AMD (preferred) or stable exudative AMD,

- Monocular and should be implanted in the better seeing eye,

- Patient willingness,

- If the patient is a candidate for cataract surgery, implantation should be done 3 months after the surgery.

### Contraindications

- Visual acuity <0.1,

- Exudative AMD, aphakia,

- Zonular weakness, pseudoexfoliation, or lens subluxation,

- Photopic pupil diameter <2.5 mm, narrow angle (< grade 2),

- Chronic uveitis, rubeosis iridis, retinal detachment, severe ocular trauma,

- Progressive glaucoma, extensive visual field defect,

- Conditions such as corneal diseases if the fundus cannot be clearly visualized.

### Telescopic Contact Lenses

Research on telescopic contact lenses is also currently ongoing. A telescopic lens that allows shifting between normal and magnified vision with three-dimensional glasses and electrical polarization was first designed experimentally in 2013 by Tremblay et al.^28^ based on an optomechanical eye model. It provided 2.8X magnification.

**Figure 6:**
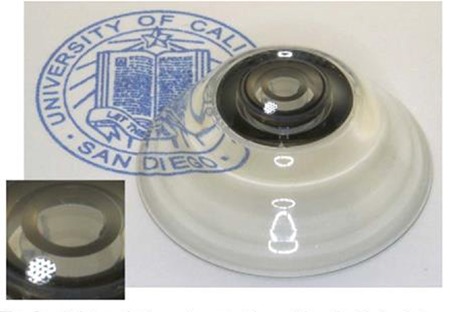
Telescopic scleral contact lens

**Figure 7:**
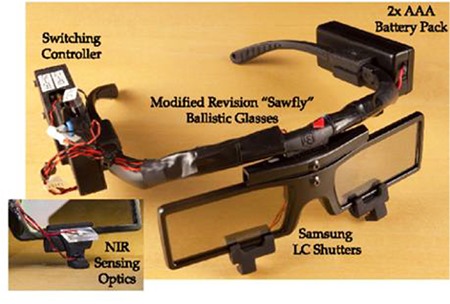
The glasses worn with telescopic scleral contact lenses

Designed as 1.6 mm-thick scleral contact lenses, corneal oxygenation was a problem with the long-term use of these telescopic contact lenses, and further research to solve this problem was recommended.^29^ A later study mentions work on a scleral telescopic contact lens in which polarization is switched by blinking, thereby allowing a shift between normal and magnified vision (Figure 6).^30^ This telescopic system is used in combination with battery-operated glasses that use LCD technology to complement the contact lens (Figure 7).^30^

In addition to their psychosocial benefits, telescopic contact lenses have advantages such as lower weight and cost and wider visual field compared to conventional spectacle-mounted telescopes.^31^

### Argus II Epiretinal Prosthesis (Bionic Eye)

This model is used in patients with severe photoreceptor cell loss. Although both retinitis pigmentosa and AMD patients experience photoreceptor cell loss, currently the primary indication for the Argus is advanced retinitis pigmentosa. It is the first and only retinal prosthesis approved by the Food and Drug Administration, and directly stimulates internal retinal cells. The Argus II delivers electrical stimulation to the retinal ganglion cells to produce spots of light called phosphenes. Patients learn to interpret these visual perceptions, thus providing some level of vision.^32,33^ The vision provided is artificial vision. This surgery was performed with endoscopic assistance for the first time in Turkey and the world by Ozmert E and Demirel S^34^ at Ankara University.

**Figure 8:**
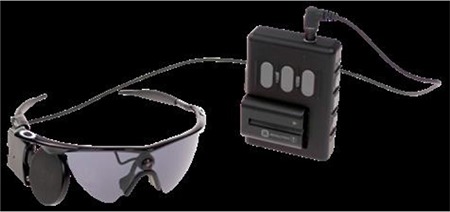
Argus II, extraocular part (http://secondsight.com/photos.html. Accessed on 08.18.2018)

**Figure 9:**
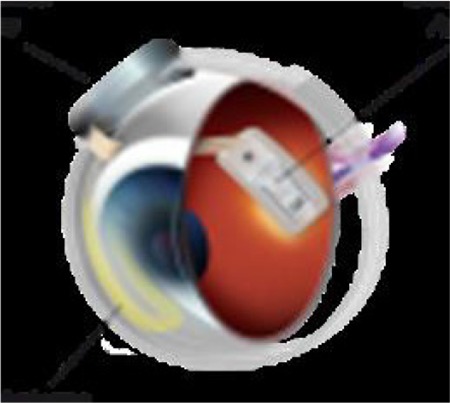
Argus II, intraocular part (http://secondsight.com/photos.html. Accessed on 08.18.2018)

**Figure 10:**
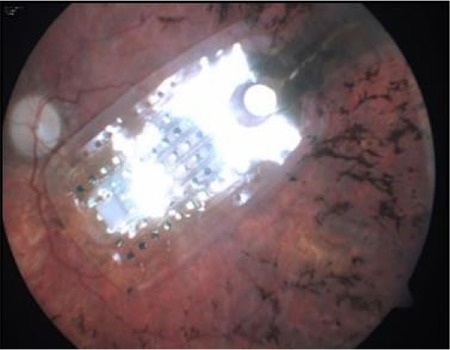
The electrode array of the Argus epiretinal prosthesis when implanted on the macula (from the archive of Prof. Emin Özmert, MD)

**Figure 11:**
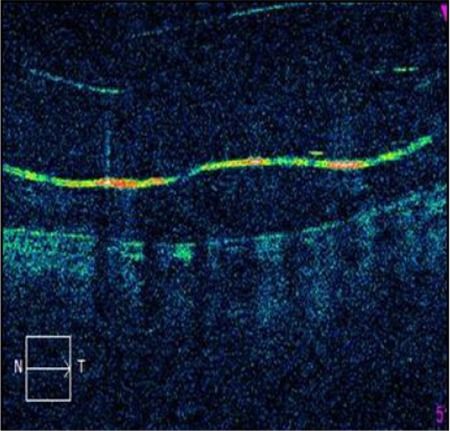
Appearance of shadows of the electrodes implanted on the macula in optical coherence tomography (from the archive of Prof. Emin Özmert, MD)

The Argus II epiretinal prosthesis has two parts, intraocular and extraocular. The extraocular part consists of a pair of glasses with a camera in the middle, a transmitter, and a video processing unit, and can be worn and removed independent of the intraocular part (Figure 8). The intraocular part consists of an array of 60 electrodes, receiver coil, electronics case, and scleral band (Figure 9). The electrode array is placed epiretinally on the macula through a vitrectomy and screwed to the retina (Figures 10 and 11).^35^

### How do Patients See with the Argus Epiretinal Prosthesis?

The camera in the glasses captures images and transmits the information to the VPU, which is worn at the waist. The VPU converts images into electronic signals which it sends to the transmitters on the glasses. Electronic signals are sent to the receiver in the eye. The data are transmitted to the electrode array implanted in the retina via a thin cable. The optic nerves then send these electrical signals to the brain. Currently the image is black and white and is artificial vision, but studies are being conducted on how to produce color vision.

**Figure 12:**
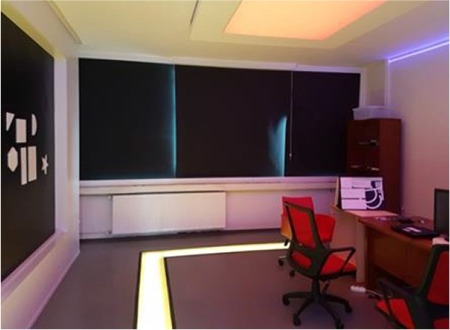
Argus rehabilitation room (from the Vision Research and Low Vision Rehabilitation Center)

**Figure 13:**
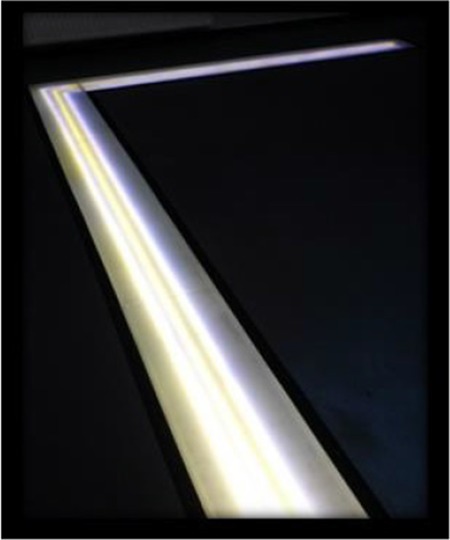
Illuminated path designed for the walking exercises of patients undergoing Argus rehabilitation (from the Vision Research and Low Vision Rehabilitation Center)

Following implantation, patients require approximately 1 year of rehabilitative support to adapt to this new system of artificial vision. The Argus rehabilitation room in our center is specially designed for the adaptation exercises and training done during the rehabilitation period (Figures 12 and 13).

### Indications for ARGUS II Epiretinal Prosthesis

- Age 25 years and older,

- Severe outer retinal cell destruction (late stage retinitis pigmentosa, geographic atrophy),

- Axial length 20-26 mm,

- Has light perception and pupillary light reflex in camera flash test,

- Has vision experience, has previously seen shapes,

- Has realistic expectations,

- Patient and relative compliance with rehabilitation.

### Contraindications for ARGUS II Epiretinal Prosthesis

- Optic nerve disease,

- Thin conjunctiva (failed surgery),

- Severe ocular pruritus,

- Inability to receive general anesthesia,

- Severe macular edema, macular scar, severe retinal thinning, posterior staphyloma,

- Severe strabismus and nystagmus,

- Neurologic and psychiatric illnesses.

In the Functional Low-Vision Observer Rated Assessment Study, 26 patients that underwent Argus II Retinal Prosthesis implantation were monitored for 18-44 months (mean 36 months) and a significant increase was reported in the rate of their completion of vision-related tasks when the device was on compared to when it was off.^36^

The Argus II Epiretinal prosthesis has been found to provide the following benefits: seeing capital letters, reading short words (best recorded visual acuity: 20/1262), discerning the direction of movements, discerning orientation and being able to move, increased mobility, ability to act independently, and increased quality of life.^35^

### BrainPort

**Figure 14:**
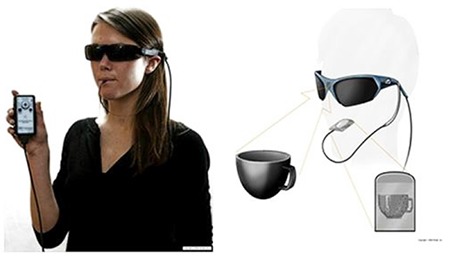
BrainPort usage (Courtesy of Wicab, Inc.)

This device also provides artificial vision, and the patient must have previously experienced vision. In the BrainPort, a 2.5-cm camera mounted on glasses sends the image it records to a handheld remote-control unit and the image is converted into a low-resolution black and white photo. This photo is then transmitted to the tongue through a thin tube containing hundreds of electrodes and the user can feel the shape and movement projected on their tongue. By visualizing the sensation on the tongue, the person learns to see the photograph (Figure 14).^37,38^

### Stem Cell Therapy in Low Vision Patients

Stem cells are progenitor cells, meaning they possess the abilities of self-renewal and differentiation into mature cells. Stem cell therapy aims to replace diseased retinal cells with new retinal cells that grow from stem cells. Stems cells have properties and functions such as high proliferative capacity, immune system regulation, secretion of neurotrophic factors, and an antiapoptotic effect on neurons. Stem cell therapy is promising for degenerative diseases of the retina such as retinitis pigmentosa, Stargardt macular dystrophy, and AMD. The outcomes of phase I and II trials have been quite successful, and no systemic side effects have been observed.^39^

Embryonic stem cells are pluripotent, but their use is unethical and prohibited by the health ministry in Turkey. Adult mesenchymal stem cells are most commonly used in patients with low vision. These cells are multipotent. Adipose tissue and bone marrow are the most preferred sources. In addition, induced pluripotent stem cells, umbilical cord blood stem cells, and amniotic fluid stem cells also have areas of application in various diseases.^40^

In patients with low vision, stem cell therapy can be used in patients over 18 years of age who have a degenerative retinal disease and is applied to the poorer seeing eye. Subretinal mesenchymal stem cell injection is performed with total vitrectomy. The procedure can be repeated when the stem cells lose functionality. The purpose is to preserve the visual field and prevent disease progression. It is not necessary to wait for a decrease in visual acuity; this treatment can be applied if visual field loss has begun. There are currently some uncertainties regarding this treatment. Controversial issues include which type of stem cell to use, at what dose, through what administration route, and at what stage of disease. In a study by Oner et al.^41^ including 11 patients with retinitis pigmentosa, only 1 of which showed improvement in electroretinogram results and significant improvement in visual acuity and visual field, the authors reported that the procedure may cause ocular complications and must be performed very carefully.

The vitreoretinal complications seen after intravitreal and subretinal stem cell injections were reported to occur less frequently with suprachoroidal administration.^42^

### Platelet-Rich Plasma Therapy and Electrical Stimulation in Patients with Low Vision

In PRP therapy, blood from the patient is centrifuged to obtain a platelet concentration 2-4 times that in the blood. PRP therapy is an autologous method. Injection enables growth factors produced by platelets (NGF, BDNF, BFGF, IL-6) to maintain the viability of the retinal photoreceptor cells. The goal is to maintain the viability of dormant cells. Treatment aims to slow disease progression, expand the visual field, and increase visual acuity. In a study of 71 eyes of 48 patients with retinitis pigmentosa, of which 49 eyes received autologous PRP via sub-Tenon’s injection, statistically significant improvements in multifocal electroretinogram values and microperimetry readings were reported and positive visual outcomes were also observed. The patients were monitored for 1 year. Long-term outcomes are unknown.^43^ Further studies with longer follow-up periods are needed to determine the duration of effect and optimal frequency of administration.

### Transcorneal Electrical Stimulation - Okuvision

**Figure 15:**
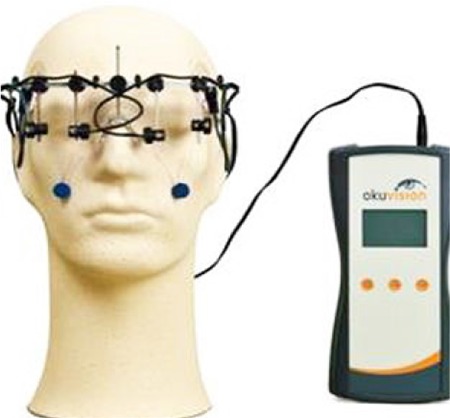
Implementation of transcorneal electrical stimulation - okuvision (https://www.retina-implant.de/en/. Accessed on 08.18.2018. Reproduced with permission from Retina Implant AG)

Low-dose electrical stimulation is delivered to retinal cells. It can be performed in conjunction with PRP injection. Treatment aims to protect retinal cells and prevent further vision loss with the release of neurotrophic growth factors. It is performed transcorneally. An electrode is placed in the cornea (Figure 15). The procedure lasts 30 minutes, with sessions performed once a week for 6-8 weeks. Some problems may be arise due to contact with the cornea. Bittner AK and Segeer K^44^ reported significant improvements in visual acuity, rapid contrast sensitivity function, and/or Goldmann visual field test results in 4 of 7 patients in the retinitis pigmentosa patient group who underwent 6 weeks of transcorneal electrical stimulation (TES) therapy. Three of these 4 patients were monitored for 29-35 months and no regression in the achieved improvements was observed.

### Transcranial Electromagnetic Stimulation - Magnovision

The aim is to stop the apoptosis cascade and reduce cell death. Magnovision uses magnetic stimulation; however, unlike the electrical stimulation in TES, the stimulus is not applied to the retina locally, but is delivered centrally. While TES involves contact with the cornea, Magnovision does not. It can be performed in conjunction with PRP injection. The goal of Magnovision combined with PRP therapy is revival of dormant photoreceptors and expansion of the visual field.

### Gene Therapy in Low Vision Patients

This treatment modality involves a genome that encodes a functional product that exerts its effect in another cell, with or without being added to that cell’s genome. The genes are carried by vectors. Adenoviruses and lentiviruses are most commonly used for this purpose. It is administered as a subretinal injection. It can be used for treating autosomal recessive and X-linked diseases. Currently, the biggest drawbacks to this method are the large number of genes that cause disease and the mutations that have occurred within the same gene.

More than 220 genes have been identified in retinal diseases. More than 160 genes and different mutations in the same gene have been identified in retinitis pigmentosa. The most studied diseases in terms of gene therapy are Leber’s congenital amaurosis and retinitis pigmentosa. The *RPE65* gene is the most studied.^45^ The roles of the *CNGA3* and *CNGB3* genes in achromatopsia and of the *ABCA4* gene in Stargardt disease are being investigated.^46^

LUXTURNA^TM^-Spark (voretigene neparvovec-rzyl) is the only drug approved by the Food and Drug Administration for use in gene therapy. It was approved for use in the treatment of hereditary retinal diseases.^47^ It can be administered as a subretinal injection. Its use is not permitted in those under the age of 1 year or over the age of 65 years.

Requirements for implementing gene therapy include a significant decrease in vision, compatibility of the target gene with the vector capacity, completed human trials involving the target gene, and the presence of intact retinal cells that can be repaired with gene therapy.

## Conclusion

There are many exciting and promising developments regarding the rehabilitation and treatment of patients with low vision. However, a patient’s age, diagnosis, education level, and sociocultural status should be considered when presenting rehabilitation and treatment options, and patients with low vision should be guided at the right age, to the right centers, and most importantly, with realistic expectations.
